# Inhibitors of Activin Receptor-like Kinase 5 Interfere with SARS-CoV-2 S-Protein Processing and Spike-Mediated Cell Fusion via Attenuation of Furin Expression

**DOI:** 10.3390/v14061308

**Published:** 2022-06-15

**Authors:** Maja C. Mezger, Carina Conzelmann, Tatjana Weil, Pascal von Maltitz, Dan P. J. Albers, Jan Münch, Thomas Stamminger, Eva-Maria Schilling

**Affiliations:** 1 Institute of Virology, Ulm University Medical Center, 89081 Ulm, Germany; maja.mezger@uni-ulm.de (M.C.M.); eva-maria.schilling@uni-ulm.de (E.-M.S.); 2 Institute of Molecular Virology, Ulm University Medical Center, 89081 Ulm, Germany; carina.conzelmann@uni-ulm.de (C.C.); tatjana.weil@uni-ulm.de (T.W.); pascal.von-maltitz@uni-ulm.de (P.v.M.); dan.albers@uni-ulm.de (D.P.J.A.); jan.muench@uni-ulm.de (J.M.)

**Keywords:** SARS-CoV-2, COVID-19, protein kinase inhibitor, ALK5, TFG-β, furin

## Abstract

Screening of a protein kinase inhibitor library identified SB431542, targeting activin receptor-like kinase 5 (ALK5), as a compound interfering with SARS-CoV-2 replication. Since ALK5 is implicated in transforming growth factor β (TGF-β) signaling and regulation of the cellular endoprotease furin, we pursued this research to clarify the role of this protein kinase for SARS-CoV-2 infection. We show that TGF-β1 induces the expression of furin in a broad spectrum of cells including Huh-7 and Calu-3 that are permissive for SARS-CoV-2. The inhibition of ALK5 by incubation with SB431542 revealed a dose-dependent downregulation of both basal and TGF-β1 induced furin expression. Furthermore, we demonstrate that the ALK5 inhibitors SB431542 and Vactosertib negatively affect the proteolytic processing of the SARS-CoV-2 Spike protein and significantly reduce spike-mediated cell–cell fusion. This correlated with an inhibitory effect of ALK5 inhibition on the production of infectious SARS-CoV-2. Altogether, our study shows that interference with ALK5 signaling attenuates SARS-CoV-2 infectivity and cell–cell spread via downregulation of furin which is most pronounced upon TGF-β stimulation. Since a TGF-β dominated cytokine storm is a hallmark of severe COVID-19, ALK5 inhibitors undergoing clinical trials might represent a potential therapy option for COVID-19.

## 1. Introduction

In late 2019, an outbreak of pneumonia in Wuhan, which was later assigned to be caused by a novel coronavirus named SARS-CoV-2, initiated an ongoing global pandemic [1,2]. Until now, COVID-19, the disease associated with SARS-CoV-2, has caused more than 6 million deaths making it one of the deadliest pandemics in human history. Beyond that, the pandemic has resulted in an immense social and economic disturbance that will probably resonate for years. Remarkably, there was a fast approval and distribution of several vaccines protecting vaccinated individuals from severe illness and hospitalization. Efficient therapeutics, however, are still urgently required due to infections of non-vaccinated individuals or break-through infections of vaccinated individuals. Moreover, the emergence of SARS-CoV-2 variants, which are more contagious and evade vaccine induced immunity, is a major concern. These so-called variants of concern (VOCs) bear several spike protein mutations that mainly decrease the immunogenicity or increase affinity to the human angiotensin-converting enzyme 2 (ACE2)-receptor and thus increase infectivity [3]. Additional mutations (P681R or P681H) located in the multibasic cleavage site have been identified in the emerging alpha, delta, and omicron variants, making those variants more transmissible [4].

Furin, belonging to the family of proprotein convertases, was identified as one of the cellular proteases required to proteolytically activate the SARS-CoV-2 spike protein. During proteolytic activation, the spike protein, which is synthesized as an inactive precursor, is converted to its active form, thus enabling membrane fusion and the subsequent delivery of viral particles into the host cell [5,6]. Cleavage of the spike protein occurs in a sequential manner at two distinct sites [7,8]. First, the multibasic S1/S2 cleavage site undergoes cleavage by furin (priming cleavage) during the biosynthesis of the spike protein [5,6]. Second, upon the binding of the spike protein to the ACE2-receptor, the S2′ site is cleaved by TMPRSS2 on the plasma membrane, thereby initiating membrane fusion and virus entry [9,10,11]. Remarkably, the expression and localization of the spike protein to the membrane of an infected host cell can induce cell–cell fusion among neighboring cells (forming multinucleated enlarged cells called syncytia), which has been hypothesized to facilitate the transfer of viral genomes via cell-to-cell transmission [12,13,14]. Moreover, the cell-to-cell transmission of viruses has recently been reported to allow for efficient spread as this route, in contrast to cell-free infection, is refractory to inhibition by neutralizing antibodies or the convalescent sera of COVID-19 patients [14]. In accordance, the presence of infected syncytial pneumocytes was documented in the lungs of patients with severe COVID-19 [15,16,17]. An additional study could further prove that emerging VOCs with increased pathogenicity are associated with enhanced syncytia formation [18].

Furthermore, it has been reported that the multibasic cleavage site is not only required for efficient cell–cell fusion and syncytia formation but also for entry into human lung cells [5,6,19,20]. Moreover, studies utilizing different animal models could show that the multibasic cleavage site is indispensable for SARS-CoV-2 infection, pathogenesis, and transmission [21,22,23,24]. While natural polymorphisms that lose the multibasic cleavage site and mutations of basic arginines are very rare, non-arginine residues continue to be permissive to optimization thus suggesting that the multibasic cleavage site is under a strong selective pressure in humans [22,25,26,27].

Consequently, furin is discussed as a promising target for antiviral interventions [28,29]. Two furin inhibitors, decanoyl-RVKR-CMK (CMK) and naphthofluorescein, have already been demonstrated to be effective against SARS-CoV-2 in a cell culture model as both can suppress spike protein cleavage and syncytia formation as well as virus production and cytopathic effects [6,19]. In addition to a direct furin inhibition, the transcriptional regulation of furin expression could be another approach. It has been demonstrated that transforming growth factor (TGF)-β1, through the activation of the transcription factor Smad2, is able to regulate furin gene expression in rat synovial and NRK-49F fibroblastic cells, in mink lung epithelial cells (Mv1Lu), in the human liver cell line HepG2, and in primary human bronchial epithelial cells [30,31,32,33]. The activation of Smad2 occurs via phosphorylation by the activin receptor-like kinase 5 (ALK5) upon the binding of TGF-ß1 [34,35]. In line with this, it has been shown that an ALK5 inhibitor is able to prevent TGFß1-mediated furin expression in primary rat brain endothelial cells [36]. In contrast to the direct furin inhibitors CMK and naphthofluorescein, several ALK5-targeting drugs are currently undergoing clinical trials to evaluate their antitumor activity, since ALK5 is an attractive target for intervention in TGF-ß signaling due to its drug ability as well as its centrality and specificity in the pathway [37].

As viruses utilize the host cell for replication, cellular proteins represent an attractive target for antiviral interventions. Cellular protein kinases have already been suggested as possible mediators of multiple viral infections, including life-threatening coronaviruses like SARS-CoV-1, MERS-CoV, and SARS-CoV-2 [38,39]. Furthermore, protein kinases emerge as an important group of drug targets accounting for 20–30% of the drug discovery programs of major pharmaceutical companies [38]. Therefore, the repurposing of drugs targeting kinases could be a sophisticated time- and cost-saving strategy. To identify such inhibitors, we screened a library consisting of approximately 160 selective and non-selective kinase inhibitors for compounds suppressing authentic SARS-CoV-2 infection. This approach resulted in the identification of SB431542, which is an inhibitor of ALK5, a kinase that transcriptionally regulates the expression of the spike-activating protease furin [31,32,36]. We show that the ALK5 inhibitor negatively affects the fusogenic potential of the spike protein by decreasing the furin expression and spike protein cleavage in spike-expressing cells. This was not only true under physiological conditions but also under TGF-ß1 stimulation, which may reflect the SARS-CoV-2-induced cytokine storm and might serve as an additional trigger for furin expression and spike cleavage [40,41,42,43]. Furthermore, we showed that treatment with the novel inhibitor SB431542 reduced the amount of newly produced infectious viral particles in an experimental set-up with authentic SARS-CoV-2, presenting further evidence for a reduced cleavage efficiency of the spike due to the inhibition of furin. Taken together, we present ALK5 as a novel target for antiviral interventions that might aim at reducing the cell-to-cell transmission of SARS-CoV-2 variants with optimized multibasic cleavage sites.

## 2. Materials and Methods

### 2.1. Cells and Viruses

HEK293T, HeLa, Vero E6, and Caco-2 cells were cultivated in Dulbecco’s minimal essential medium (DMEM) (Thermo Fisher Scientific, Waltham, MA, USA) supplemented with 10% fetal calf serum (FCS) and penicillin-streptomycin (Sigma-Aldrich, St. Louis, MO, USA). Huh-7 cells were cultivated in RPMI 1640 medium (Thermo Fisher Scientific, Waltham, MA, USA) supplemented with 10% FCS, penicillin-streptomycin (Sigma-Aldrich, St. Louis, MO, USA), and GlutaMAX (Thermo Fisher Scientific, Waltham, MA, USA). Calu-3 cells were cultivated in minimal essential medium (MEM) (Thermo Fisher Scientific, Waltham, MA, USA) supplemented with 12.5% FCS, penicillin-streptomycin (Sigma-Aldrich, St. Louis, MO, USA), non-essential amino acids (Thermo Fisher Scientific, Waltham, MA, USA), and sodium pyruvate (Thermo Fisher Scientific, Waltham, MA, USA).

SARS-CoV-2 variant of concern (VOC) Alpha was obtained from the European Virus Archive global (hCoV-19/Netherlands/NH-RIVM-20432/2020, #014V-04031). SARS-CoV-2 Delta isolate was kindly provided by Prof. Hendrik Streek, Bonn. Virus was propagated by inoculation of 70% confluent TMPRSS2-expressing Vero E6 or Caco-2 cells. After 3–4 days, supernatant was harvested. To this end, supernatants were centrifuged 5 min at 1000× *g* to remove cellular debris, and then aliquoted and stored at −80 °C as virus stocks. Infectious virus titer was determined as plaque forming units (PFU) which were used for multiplicity of infection (MOI) calculation.

### 2.2. Substances

The kinase screening library was purchased as 10 mM solution in DMSO from Cayman Chemicals (Ann Arbor, MI, USA). SB431542 (S1067) and Vactosertib (S7530), both provided by Selleckchem (Houston, TX, USA), as well as Decanoyl-RVKR-CMK (150113-99-8), provided by Santa Cruz Biotechnolgy (Dallas, TX, USA), were dissolved in DMSO for our experiments. Recombinant human TGF-ß1 (240-B/CF) was purchased from R&D systems (Minneapolis, MN, USA) and reconstituted at 20 µg/mL in sterile 4 mM HCl containing 0.1% bovine serum albumin.

### 2.3. Plasmids

A plasmid encoding the SARS-CoV-2 Wuhan-Hu-1 wildtype (wt) spike (pCAGGS_Spike_wt) was a kind gift from Florian Krammer [44]. Stefan Pöhlmann and Markus Hoffmann kindly provided the following plasmids encoding spike proteins of different variants, which have been described elsewhere [45,46,47]:

- D614G: pCG1_SARS-2-Sdel18_D614G (D614G);
- Alpha: pCG1_SARS-2-Sdel18_DG614G + VOC-202012_01 (UK variant);
- Beta: pCG1_SARS-2-Sdel18_DG614G + SA_501-V2new (South Africa variant);
- Gamma: pCG1_SARS-2-Sdel18_DG614G + Brasil;
- Kappa: pCG1_SARS-2-Sdel18_D614G (IND) B.1.617.1 (kappa);
- Omicron BA.1: pCG1_SARS-2-Sdel18 (B.1.1.529/Omicron BA.1).

The expression plasmid for SARS-CoV-2 Delta variant was a kind gift from Beatrice Hahn [48,49].

### 2.4. Nucleocapsid Protein In-Cell ELISA

To assess SARS-CoV-2 infection rates, an in-cell ELISA targeting SARS-CoV-2 nucleocapsid was applied. Briefly, 50,000 Caco-2 cells were treated with the compounds of interest and infected with SARS-CoV-2 VOC Alpha at a MOI of 0.0005. Two days later, cells were fixed by incubating in 4% paraformaldehyde (PFA) for 30 min and permeabilized by incubation with 0.1% Triton-X for 5 min. After washing once with PBS, cells were stained with 1:5000 diluted anti-nucleocapsid antibody (40143-MM05, Sino Biological, Beijing, China) in antibody buffer (10% FCS and 0.3% Tween 20 in PBS) for 1 h at 37 °C. After two washes with 0.3% Tween 20 in PBS, the secondary HRP-conjugated antibody (#A16066, Thermo Fisher Scientific, Waltham, MA, USA) (1:15,000) was incubated for 1 h at 37 °C. Cells were washed three times with 0.3% Tween 20 in PBS, TMB peroxidase substrate (#52-00-04, SeraCare, Milford, MA, USA) was added for 5 min, and the reaction was stopped using 0.5 M H_2_SO_4_. The optical density (OD) was recorded at 450 nm using the Asys Expert 96 UV microplate reader (Biochrom, Cambridge, UK) with DigiRead 1.26 software. Values were corrected for the background signal derived from uninfected cells and untreated infection controls were set to 100% infection.

For transfer experiments, 4 × 10^4^ Calu-3 cells were seeded one day before treatment. After 24 h cells were treated with compounds of interest, titrated in PBS, and incubated for an additional 24 h. Afterwards cells were infected with SARS-CoV-2 VOC Delta at a MOI of 0.0005. Two days post infection, supernatants were transferred onto 7 × 10^4^ fresh Calu-3 cells seeded the day before and incubated for an additional 24 h at 37 °C before in-cell ELISA was performed as described previously. Additionally, in-cell ELISA was also performed on initial infection plates. After fixation all plates were stained as described above. For analysis, background was subtracted, and untreated infection controls were set to 100% infection.

### 2.5. CellTiter-Glo Luminescent Cell Viability Assay

The effect of the compounds on the metabolic activity of the cells was analyzed using the CellTiter-Glo^®^ Luminescent Cell Viability Assay (#G7571, Promega, Madison, WI, USA) according to the manufacturer’s instructions. Metabolic activity was examined under conditions corresponding to the respective infection assays. Briefly, medium was removed from the culture after two days of incubation and 50% substrate reagent in PBS was added. After 10 min, luminescence of the samples was measured in an Orion II Microplate Luminometer (Titertek Berthold, Pforzheim, Germany).

### 2.6. Transfection

HEK293T cells were transfected with plasmid DNA by utilizing the Lipofectamin 2000 transfection reagent according to instructions of the manufacturer (Thermo Fisher Scientific, Waltham, MA, USA). For this, 7 × 10^5^ HEK293T cells were seeded into six-well dishes. One day after seeding, cells were transfected with plasmid DNA coding for SARS-CoV-2 spike variants (1 µg) and/or eGFP (200 ng). At about five hours later, fresh medium was provided. Cells were harvested 48 h post transfection and utilized for immuno-blotting or cell–cell fusion assay. In case a substance treatment was required for the respective experiment, cells were incubated overnight with the substances before the transfection took place. Additionally, substances were renewed when cells were provided with fresh medium after transfection.

### 2.7. Immunoblotting

Transfected and/or substance-treated cells were harvested and lysed for 20 min at 4 °C by the addition of RIPA buffer (50 mM Tris/HCl pH 7.4, 150 mM NaCl, 1% NP40, 0.5% sodium deoxycholate, 0.1% sodium dodecyl sulfate, 2 μg/mL of aprotinin, 2 μg/mL of leupeptin, and 2 μg/mL of pepstatin). After centrifugation, the supernatant was diluted in sodium dodecyl sulfate-Laemmli buffer and boiled at 95 °C for 10 min. Proteins were separated by SDS-polyacrylamide gel electrophoresis (SDS-PAGE) on 8% polyacrylamide gels and transferred onto PVDF membranes (Bio-Rad, Hercules, CA, USA), followed by chemiluminescence detection using a FUSION FX7 imaging system (Vilber Lourmat Deutschland GmbH, Eberhardzell, Germany). The following antibodies were used: rabbit polyclonal antibody α-furin NB100-1903 (Novus Biologicals, Littleton, CO, USA), mouse monoclonal antibody α-Spike S2 1A9 (Thermo Fisher Scientific, Waltham, MA, USA), and mouse monoclonal antibody α-ß-actin AC-15 (Sigma-Aldrich, St. Louis, MO, USA).

### 2.8. Cell–Cell Fusion Assay

HEK293T cells were transfected and substance-treated as described above. At 48 h post transfection, cells were rinsed and collected in a reaction tube. After a centrifugation step for 5 min at 4000 rpm, HEK293T cells were resuspended in PBS and subsequently counted by utilizing a Neubauer counting chamber. A total of 3 × 10^5^ HEK293T cells were added onto 4 × 10^5^ Huh-7 cells, which were seeded 24 h before on glass coverslips. Two hours later, cells were washed twice with PBS. Subsequently, cells were fixed with a 4% paraformaldehyde solution for 10 min at room temperature (RT) and then washed twice. Permeabilization of cells was achieved by incubation with 0.2% Triton X-100 in PBS on ice for 20 min. Cells were washed again with PBS over a time period of 5 min and incubated with mouse monoclonal antibody α-Spike S2 1A9 (Thermo Fisher Scientific, Waltham, MA, USA) diluted in PBS-1% FCS for 30 min at 37 °C. Excessive antibodies were removed by washing four times with PBS, followed by incubation with an Alexa Fluor 555-coupled goat-α-mouse secondary antibody diluted in PBS-1% FCS for 30 min at 37 °C. The cells were mounted using the DAPI-containing Vectashield mounting medium (VECTOR LABORATORIES, Burlingame, CA, USA) and analyzed using an Axio-Observer.Z1 fluorescence microscope (Carl Zeiss Microscopy GmbH, Oberkochen, Germany) with 469/38 nm and 555/30 nm LED sources.

## 3. Results

### 3.1. Identification of an ALK5 Kinase Inhibitor That Supresses SARS-CoV-2 Infection

**Figure 1:**
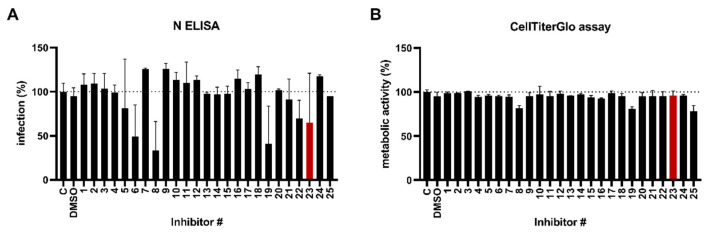
Screening of kinase inhibitors against SARS-CoV-2. (**A**) Compounds were prediluted in medium and added onto Caco-2 cells (final conc. 1 µM). Afterwards, cells were infected with SARS-CoV-2 Alpha (MOI of 0.0005) and 2 days later, infection was quantified by an in-cell ELISA. Values were corrected for the background signal derived from uninfected cells and untreated controls were set to 100% infection. (**B**) In parallel, a viability assay was performed under conditions corresponding to the respective infection assay. Metabolic activity of the cells was assessed 2 days later using the CellTiter-Glo^®^ Luminescent Cell Viability Assay. Untreated controls were set to 100% viability. Shown are mean values ± SD from one experiment performed in triplicates. Red bar indicates compound of interest SB431542 (Inhibitor # 23), C: control.

To identify the kinases required for the life cycle of SARS-CoV-2, we made use of a kinase screening library that offers an expansive coverage, targeting more than 70 distinct kinases and kinase families, as well as numerous additional kinase isoforms and individual kinases within target families (Supplementary Table S1). For this, kinase inhibitors were added onto Caco-2 cells at a final concentration of 1 µM, followed by infection with SARS-CoV-2 Alpha. Two days later, infection rates were quantified by an in-cell ELISA that enzymatically quantifies nucleocapsid protein [50]. In parallel, a cell viability assay was performed. This approach enabled the identification of several kinase inhibitors that were antiviral without being cytotoxic (# 6, 8, 19, 22, 23, 29, 36, 38, 139, 148, 150, see Table S1 for designation) (Figure 1 and Supplementary Figure S1). Among them, we found inhibitors that are already proposed to negatively affect the SARS-CoV-2 life cycle (# 8, 22, 29, 38, 139) and/or to target kinases or even kinase signaling pathways already known to be utilized by SARS-CoV-2, namely receptor tyrosine kinases (# 22, 148), GSK3 (# 29), and components of the mTOR pathway (# 8, 19, 36) [51,52,53,54,55,56,57,58]. In addition, inhibitor # 23 (SB431542) reduced infection to 65% in comparison to the untreated infection control. SB431542 has been identified as a potent and selective inhibitor of the transforming growth factor-β (TGF-β) type I receptor/ALK5 [58,59]. It has been demonstrated to inhibit ALK5 and also the activating type I receptor ALK4 and the nodal type I receptor ALK7, which are highly related to ALK5 in their kinase domains, but it has no effect on the other more divergent ALK family members that recognize bone morphogenetic proteins (BMPs) [59]. Consistently, SB431542 has been shown to act as a selective inhibitor of endogenous activity and TGF-β signaling but has no effect on BMP signaling. Furthermore, no effects on the components of the ERK, JNK, or p38MAP kinase pathways could be observed [59]. As ALK5 is described as a kinase that transcriptionally regulates, in dependence of TGF-ß1, the expression of the spike-activating protein furin, we inquired to clarify the role of ALK5 for SARS-CoV-2 infection [31,32,36]. Other identified kinase inhibitors are currently under investigation.

### 3.2. TGF-ß1 Induces Furin Expression in Cells Permissive and Non-Permissive for SARS-CoV-2 Infection

**Figure 2:**
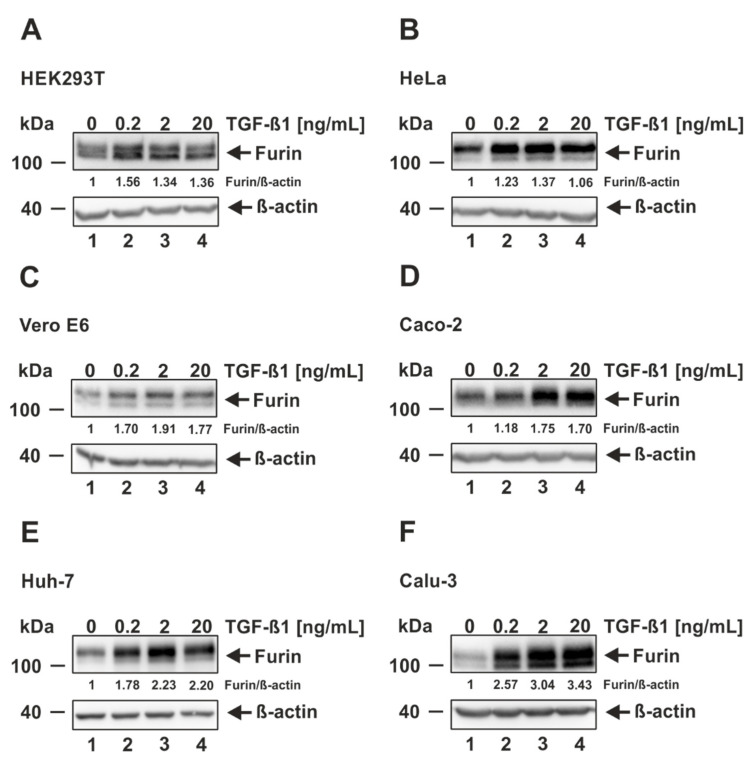
Induction of furin expression by TGF-ß1. (**A**) HEK293T, (**B**) HeLa, (**C**) Vero E6, (**D**) Caco-2, (**E**) Huh-7, and (**F**) Calu-3 were incubated for 18 h with indicated concentrations of TGF-ß1 or the solvent control 4 mM HCl 0.1% bovine serum albumin (0 ng/mL TGF-ß1). Western blot analyses were performed to detect furin and ß-actin. Protein levels of furin and ß-actin were quantified via densitometric analyses utilizing the Image Lab Software. Protein levels without the addition of TGF-ß1 (0 ng/mL) were set to 1.

To date, TGF-ß1-induced furin expression has been shown only for a small subset of cells including the human liver cell line HepG2 and primary human bronchial epithelial cells [30,31,32,33]. We therefore decided to investigate more cell lines. To this end, four SARS-CoV-2 permissive (Vero E6, Caco-2, Huh-7, and Calu-3) and two non-permissive (HEK293T and HeLa) cell lines were chosen to investigate furin expression after TGF-ß1 treatment. For this purpose, the cell lines were treated for 18 h with increasing amounts of TGF-ß1. Thereafter, cells were harvested, lysed, and analyzed via SDS-PAGE and Western blotting by utilizing an antibody directed against furin. Subsequent quantification via densitometric analyses revealed that all cell lines responded to TGF-ß1 stimulation with the upregulation of furin thereby clearly exceeding the basal protein expression (Figure 2). While some cell lines displayed maximum fold inductions below 2 (HeLa: 1.37; HEK293T: 1.56; Caco-2: 1.75; Vero E6: 1.91) (Figure 2A–D), the furin levels of Huh-7 and especially Calu-3 were highly stimulated by TGF-ß1 in a dose-dependent manner reaching fold inductions of 2.23 and 3.43, respectively (Figure 2E,F). Interestingly, the maximum effects of TGF-ß1 were observed at quite different concentrations. HEK293T cells displayed the highest furin expression at 0.2 ng/mL TGF-ß1, which is the lowest concentration utilized, whereas HeLa, Vero E6, and Caco-2 reached furin peak levels at a middle concentration of 2 ng/mL. Strikingly, the furin expression in Huh-7 and Calu-3 was even augmented until the highest concentration of 20 ng/mL TGF-ß1. Altogether, all tested cell lines responded to TGF-ß1 stimulation with the expression of furin; however, displaying a different behaviour concerning the maximum amount of furin and the TGF-ß1 concentration was required to reach this.

### 3.3. ALK5 Inhibition Decreases Furin Expression in Cells Permissive and Non-Permissive for SARS-CoV-2 Infection

**Figure 3:**
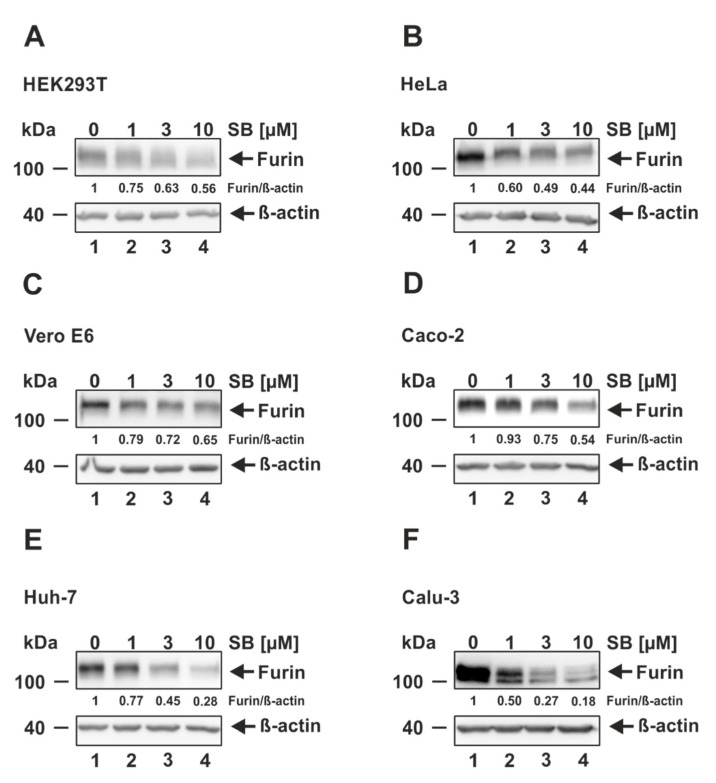
Decrease of furin expression by the ALK5 inhibitor SB431542. (**A**) HEK293T, (**B**) HeLa, (**C**) Vero E6, (**D**) CaCo-2, (**E**) Huh-7, and (**F**) Calu-3 were incubated for 24 h with the indicated concentrations of SB431542 (SB) or the solvent control DMSO (0 µM). Western Blot analyses were performed to detect furin and ß-actin. Protein levels of furin and ß-actin were quantified via densitometric analyses utilizing the Image Lab Software. Protein levels without SB (0 µM) were set to 1.

Having shown that HEK293T, HeLa, Vero E6, Caco-2, Huh-7, and Calu-3 respond to TGF-ß1 stimulation with increased furin expression, the question arose whether ALK5 inhibition, on the contrary, prevents furin expression. To answer this question, we treated all cell lines with increasing concentrations of the ALK5 inhibitor SB431542, and 24 h later, analyzed furin protein levels again by Western blotting. We detected a dose-dependent decrease of furin expression in all tested cell lines as demonstrated by densitometric analyses (Figure 3). HEK293T, HeLa, Vero E6, and Caco-2 displayed an approximately two-fold reduction of furin protein levels at the highest SB431542 concentration (10 µM) to 0.56, 0.44, 0.65, and 0.54, respectively, relative to the solvent-treated control (Figure 3A–D). Intriguingly, a much stronger effect was observed when treating Huh-7 and Calu-3 with SB431542. At the highest inhibitor concentration, we could detect a reduction of furin expression to 0.28 and 0.18 in Huh-7 and Calu-3, respectively, suggesting that furin expression in those cell lines is highly dependent on ALK5 (Figure 3E,F). Collectively, our data strongly indicate that furin expression is regulated by ALK5 in all the tested cell lines. However, the highest dependence on ALK5 signaling was observed in the SARS-CoV-2 permissive cell lines Huh-7 and Calu-3.

### 3.4. ALK5 Inhibition Prevents TGF-ß1-Induced Furin Expression

**Figure 4:**
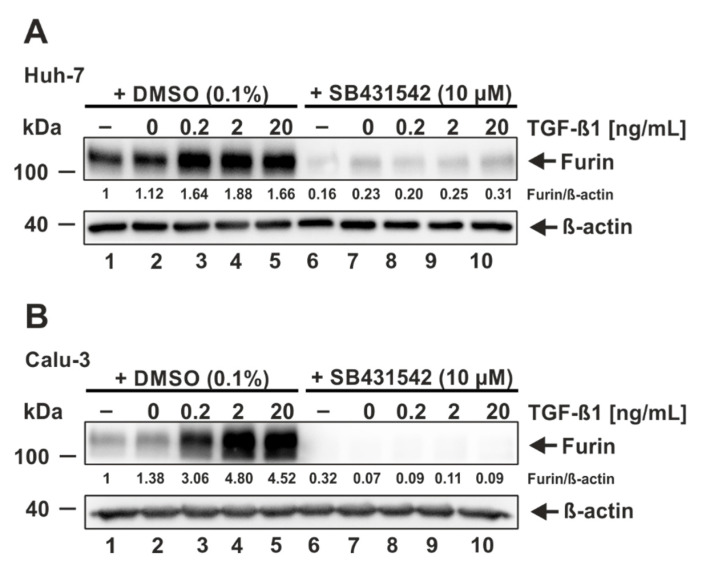
Prevention of TGF-ß1-induced furin expression by the ALK5 inhibitor SB431542. (**A**) Huh-7 and (**B**) Calu-3 were incubated for 24 h with 10 µM SB431542 or the solvent control DMSO and subsequently treated for 18 h with the indicated concentrations of TGF-ß1, the solvent control 4 mM HCl 0.1% bovine serum albumin (0 ng/mL), or were left untreated (−). Western Blot analyses were performed to detect furin and ß-actin. Protein levels of furin and ß-actin were quantified via densitometric analyses utilizing the Image Lab Software. Protein levels without the addition of SB431542 and TGF-ß1 were set to 1.

To corroborate that the observed decrease in furin expression is mediated by a direct inhibition of the TGF-ß1/ALK5 signaling cascade and not by any off-target effect, we next utilized a combination of TGF-ß1 stimulation and ALK5 inhibition. As Huh-7 and Calu-3 cells displayed the highest dependence on ALK5 signaling for furin expression and the best responsiveness to TGF-ß1 stimulation (see Figure 2 and Figure 3), we continued our analyses with these cell lines. Huh-7 and Calu-3 were treated with 10 µM SB431542 or the solvent control DMSO for 24 h and subsequently stimulated with increasing amounts of TGF-ß1 (0, 0.2, 2, and 20 ng/mL) for a time period of 18 h. Western blot and densitometric analyses again revealed a strong upregulation of furin expression following TGF-ß1 addition in DMSO-treated cells (Figure 4A,B, lanes 1–5). In contrast, the TGF-ß1-mediated upregulation of furin expression was decreased, or in the case of Calu-3 cells, even completely prevented when cells were pretreated with SB431542 (Figure 4A,B, lanes 6–10). These experiments clearly demonstrate that the inhibitor SB431542 directly acts on ALK5 to interrupt TGF-ß1-induced furin expression. Moreover, we could again detect a strong dependence on ALK5 signaling for furin expression in the lung cell line Calu-3.

### 3.5. ALK5 Inhibition Reduces Proteolytic Processing of Spike

**Figure 5:**
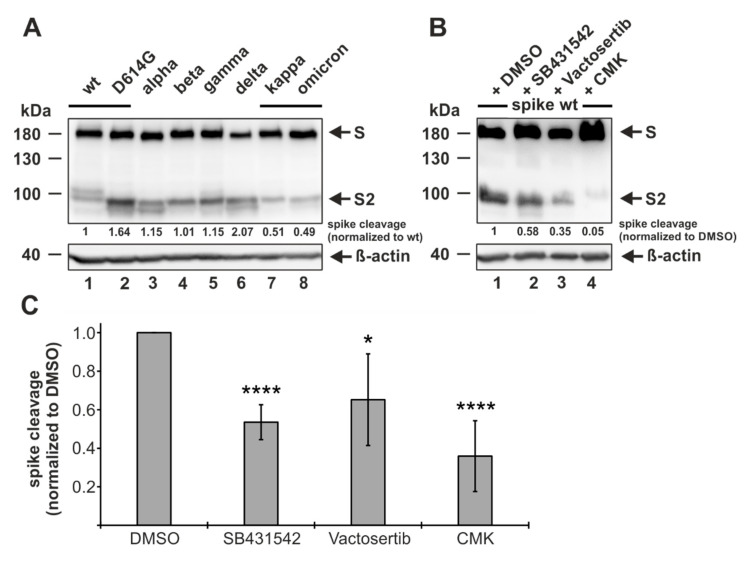
Reduction of proteolytic processing of spike by ALK5 inhibition. (**A**) HEK293T cells were transfected with the indicated constructs, and at 48 h post transfection, were harvested and analyzed by Western blotting for the S2 subunit of spike and ß-actin. Spike cleavage, normalized to the spike wildtype (wt), was determined by quantification of uncleaved spike (S) and cleaved spike (S2) via densitometric analyses utilizing the Image Lab Software. (**B**) HEK293T cells were pre-incubated with 10 µM SB431542, Vactosertib, CMK or the solvent control DMSO. 24 h later, cells were transfected with spike wt, and at 48 h post transfection, were harvested and analyzed by Western blotting for the S2 subunit of spike and ß-actin. Spike cleavage, normalized to DMSO, was determined by quantification of uncleaved spike (S) and cleaved spike (S2) via densitometric analyses utilizing the Image Lab Software. (**C**) Spike cleavage obtained by five independent experiments, conducted as in 5B, is represented by mean values ± SD. The *p*-values were calculated using two-tailed Student’s *t*-test. * *p*  ≤ 0.05; **** *p*  ≤  0.0001.

It has already been demonstrated that either directly inhibiting furin or mutating/deleting the multibasic cleavage site reduces or even prevents the proteolytic processing of the spike protein [5,6,19,20,21,23,60,61]. Thus, we speculated that utilizing the ALK5 inhibitor, which has been shown to markedly decrease furin expression, could have the same outcome. To address this, we made use of HEK293T cells that are routinely used to analyze spike processing after the transfection of plasmids coding for spike proteins [5,9,11]. Beforehand, we analyzed different SARS-CoV-2 spike variants for spike cleavage in HEK293T cells. To this end, HEK293T cells were transfected with plasmids coding for Wuhan-Hu-1 wildtype (wt) spike protein and additionally with plasmids coding for spike proteins of formerly (D614G, alpha, beta, and gamma) and currently (delta and omicron BA.1) circulating VOCs as well as of one former variant of interest (kappa). Two days later, the cells were harvested and subjected to Western blot analyses by utilizing an antibody specific for the S2 subunit of the spike protein. We observed not only equal spike protein expression levels for all SARS-CoV-2 strains but also efficient proteolytic processing. According to earlier studies, we detected one major band at 180 kDa indicative of the uncleaved spike protein and two bands at approximately 100 kDa reflecting the spike cleaved at S1/S2 and at S2′ (Figure 5A). In comparison to the wt, a strongly enhanced spike cleavage was found for delta but not for other variants, which is consistent with the findings of other groups (Figure 5A, compare lanes 1 and 6) [62,63]. In sharp contrast, the omicron BA.1 variant displayed a highly decreased spike cleavage efficiency, which is also in accordance with a recently published study (Figure 5A, compare lanes 1 and 8) [64]. After having shown that the spike is efficiently cleaved in HEK293T cell, we pursued further to investigate the effect of ALK5 inhibition on spike cleavage. To make sure that ALK5 was completely blocked before the biosynthesis of the spike protein started, we pre-incubated HEK293T cells overnight with either the solvent control DMSO, the ALK5 inhibitor SB431542, the ALK5 inhibitor Vactosertib (which is currently undergoing clinical trials), or the direct furin inhibitor CMK as a positive control. Afterwards, HEK293T cells were transfected with plasmids coding for wt spike protein. Two days later, the cells were harvested and subjected to Western blot analyses by utilizing an antibody specific for the S2 subunit of the spike protein. As seen in Figure 5B, the spike cleavage was markedly decreased in cells pre-incubated with the ALK5 inhibitors SB431542 and Vactosertib, and almost completely abolished by CMK. A quantitative evaluation of the independent experiments demonstrated that both ALK5 inhibitors reduce the amount of cleaved spike up to two-fold in comparison to the DMSO treated cells, whereas the positive control CMK leads to an almost three-fold reduction (Figure 5C).

### 3.6. ALK5 Inhibition Attenuates Spike-Mediated Cell–Cell Fusion

**Figure 6:**
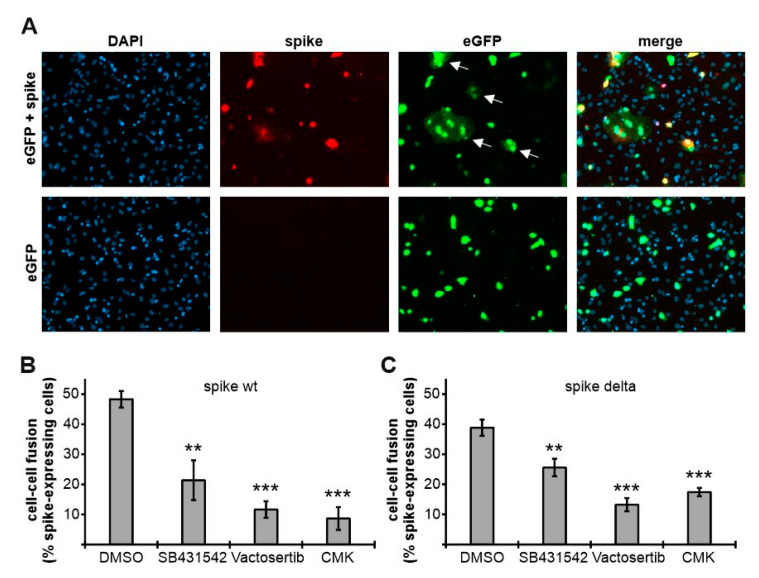
Attenuation of spike-mediated cell–cell fusion by ALK5 inhibition. (**A**) HEK293T cells transfected for two days with eGFP or both spike and eGFP were added onto Huh-7 cells. Two hours post addition, cells were analyzed by detecting the green fluorescent protein eGFP and spike protein (red) by utilizing an antibody directed against the spike S2 subunit. Nuclei were counterstained with DAPI (blue). White arrows indicate cell–cell fusion. (**B**,**C**) HEK293T cells, which were pre-treated overnight with 10 µM of the indicated substances or the solvent control DMSO, were transfected for two days with (**B**) spike wt or (**C**) spike delta and eGFP and subsequently added onto Huh-7 cells. Two hours later, cells were analyzed for cell–cell fusion (% spike-expressing cells) by detecting eGFP as well as spike. Values are derived from biological triplicates and represent mean values ± SD. The *p*-values were calculated using two-tailed Student’s *t*-test. ** *p*  ≤  0.01; *** *p*  ≤  0.001.

In previous studies, it has been shown that the priming of the cleavage at the multibasic cleavage site of spike is a prerequisite for spike-mediated cell–cell fusion [6,19,20]. To assess the role of ALK5 in spike-mediated cell–cell fusion, we adopted a protocol from the Lu group [65]. HEK293T cells, as effector cells, were transfected either with a single plasmid encoding the green fluorescent protein eGFP or with two plasmids encoding eGFP and spike. Two days later, eGFP- or eGFP/spike-expressing HEK293T cells were added onto Huh-7 cells that serve as target cells since they express high endogenous levels of the spike receptor ACE2. After two hours of co-culturing, effector and target cells were fixed, permeabilized, immunostained, and subsequently analyzed via indirect immunofluorescence analyses. As evident in the upper panel of Figure 6A, HEK293T cells expressing both eGFP and spike efficiently fused with Huh-7 cells after two hours of co-culturing (indicated by white arrows). Fused cells could be identified by their at least two-fold larger size than normal cells and the presence of multiple nuclei. In contrast, cell–cell fusion was not observed with HEK293T cells expressing eGFP alone, thereby demonstrating the dependence on the expression of spike (Figure 6A, lower panel). Next, we inquired into the question of whether ALK5 inhibitors can prevent cell–cell fusion. To answer this, we treated HEK293T cells overnight with either the solvent control DMSO, the ALK5 inhibitors SB431542 and Vactosertib, or the furin inhibitor CMK. Afterwards, HEK293T cells were transfected with plasmids coding for either spike wt or delta and eGFP. After 48 h, the transfected cells were added onto HuH-7 cells. After two hours of co-culturing, effector and target cells were again prepared for indirect immunofluorescence. Subsequently, at least 100 spike-expressing cells from randomly selected regions on the coverslips were analyzed for cell–cell fusion. By doing so, we observed 48% of spike wt-expressing HEK293T cells fused in the DMSO-treated control. In contrast, spike wt-expressing HEK293T cells treated with SB431542 and Vactosertib displayed decreased fusion rates of 21% and 12%, respectively. Similar results (9% cell–cell fusion) were obtained with spike wt-expressing HEK293T cells treated with CMK, which has already been shown to decrease spike-dependent cell–cell fusion [19] (Figure 6B). Cell–cell-fusion of spike delta-expressing HEK293T cells was equally reduced, with only 26%, 13%, and 17% for cells treated with SB431542, Vactosertib, or CMK, respectively, in comparison to 39% in the DMSO-treated control (Figure 6C). Altogether, these data clearly reveal that ALK5 inhibition attenuates spike-mediated cell–cell fusion.

### 3.7. SB431542 Reduces Infectious SARS-CoV-2 Production

**Figure 7:**
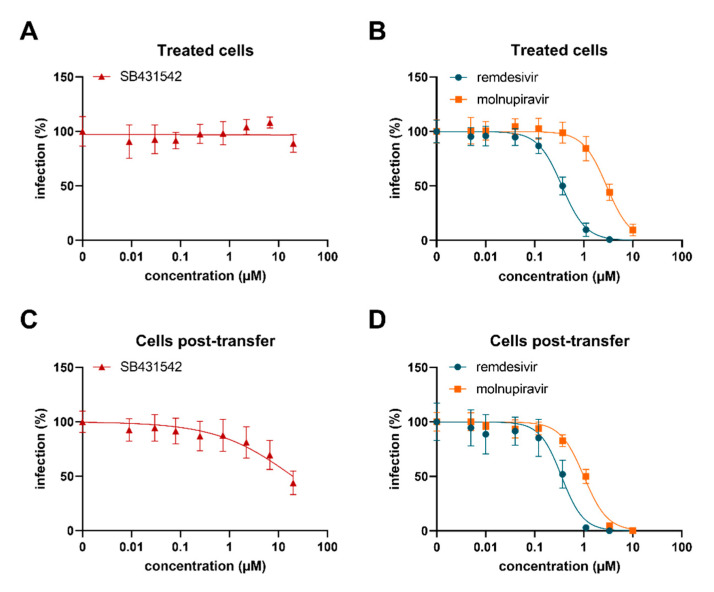
SB431542 reduces infectious virus production. (**A**,**B**) Calu-3 cells were treated with SB431542, remdesivir, or molnupiravir and infected with SARS-CoV-2 Delta at an MOI of 0.0005. Infection rates were determined two days post infection by in-cell ELISA targeting nucleocapsid. (**C**,**D**) Supernatants of (**A**,**B**) obtained two days post infection were transferred to non-infected Calu-3 cells. Infection rates were measured one day later by in-cell ELISA. Values shown represent % infection relative to infected controls containing no inhibitor. Values are derived from three independent experiments each performed in triplicates and represent mean values ± SD.

After confirming that ALK5 inhibition interferes with furin expression and therefore reduces the proteolytic processing of spike protein, we next tested whether SB431542 may affect authentic SARS-CoV-2 infection and replication. For this, Calu-3 cells were exposed to increasing concentrations of the kinase inhibitor SB431542 and the nucleoside analogue prodrugs remdesivir or molnupiravir as controls, and then infected with SARS-CoV-2 Delta. Infection rates were determined 2 days later by in-cell ELISA showing a dose-dependent decrease of N-protein positive cells in the presence of molnupiravir or remdesivir, but no inhibitory effect of SB431542 at the tested concentrations (Figure 7A,B). SB431542 was also not cytotoxic in the applied concentrations (Supplementary Figure S2). However, as SB431542 is expected to reduce the cleavage of de novo synthesized spike and thus act late in the viral life cycle, we also determined the infectivity of the progeny virus. For this, supernatants obtained at day 2 were used to inoculate fresh target cells. Infection rates were determined as described above and showed a 56% reduced infectivity as compared to the untreated control (Figure 7C,D). Thus, the ALK5 inhibitor SB431542 suppresses the production of infectious progeny viruses.

## 4. Discussion

Vaccination against SARS-CoV-2 is an important tool to prevent hospitalization and severe COVID-19. However, there are still COVID-19 cases of life-threatening diseases and deaths among older or unvaccinated people. In addition, the emergence of SARS-CoV-2 variants with spike protein mutations that confer resistance to neutralization compromise vaccine efficacy [66]. Another concern is the rapid decline of neutralizing antibodies in the sera of vaccinated people leading to the necessity of repeated booster vaccinations within short intervals. Drugs currently approved in the EU include monoclonal antibodies directed against the spike protein (Regkirona, Ronapreve, and Xevudy) or the interleukin-6 receptor (RoActemra), an interleukin-1 receptor antagonist (Kineret), and three drugs directly targeting the virus (Paxlovid, Veklury, and Lagevrio). The directly acting antivirals potently interfere with SARS-CoV-2 replication in cell culture; however, they are of limited effectiveness in advanced COVID-19 [67]. Thus, there is an additional need for novel therapy options which may include drugs that combine antiviral and immunomodulatory activities.

Screening a kinase inhibitor library for compounds that reduce SARS-CoV-2 infection/replication allowed us to identify eleven compounds with antiviral activity. Some of the inhibitors have previously been described to interfere with SARS-CoV-2 or to block kinases that are involved in the viral life cycle. For example, we found two inhibitors of the mTOR pathway which has already been shown to be exploited by SARS-CoV-2 to promote its own replication [54,55]. Furthermore, 5-iodotubercidin was very recently described as inhibiting SARS-CoV-2 RNA synthesis without providing data concerning its anti-SARS-CoV-2 activity [56]. CHIR99021 and Gö6983 have been identified to impair or reduce, respectively, N protein phosphorylation, which in the case of CHIR99021 has been shown to result in a reduction of SARS-CoV-2 infection [53]. By confirming or even extending the findings on those inhibitors, we were able to demonstrate that our screening approach is both reproducible and reliable.

Additionally, we newly identified SB431542 as an inhibitor of SARS-CoV-2. SB431542 is a potent and selective inhibitor of TGF-β type I receptor/ALK5 and its relatives, ALK4 and ALK7 [59,68]. Interestingly, ALK5 is not only required for the transcriptional regulation of furin, a main protease involved in activating the spike protein, but for other TGF-ß signaling pathways [69,70]. In this context, it is noteworthy to emphasize that enhanced levels of TGF-ß play a major role in the SARS-CoV-2 induced cytokine storm, which results in the acute respiratory distress syndrome (ARDS) associated with persistent post-COVID syndrome and high mortality [40,41,42,43,71,72]. Therefore, the blocking of TGF-ß signaling is highly considered as therapy option against severe COVID-19 [73]. Whether ALK5 inhibition is a suitable option to prevent the TGF-ß-dominated cytokine storm needs to be clarified in additional studies. Here, we assessed the role of ALK5 for furin expression, proteolytic processing of spike protein, spike-mediated cell–cell fusion, and the reduction of infectious authentic SARS-CoV-2.

So far, TGF-ß1-induced furin expression has been reported for only a small subset of cells [30,31,32,33]. Noteworthily, it has already been shown that TGF-ß1 and TGF-ß2 are both able to induce the expression of furin mRNA and protein in well-differentiated primary human bronchial epithelial cells maintained in an air–liquid interface, which is established as an experimental system for studying SARS-CoV-2 infection in vitro [33,74]. By utilizing SARS-CoV-2 permissive (Vero E6, Caco-2, Huh-7, and Calu-3) and non-permissive cell lines (HEK293T and HeLa), we were able to extend the list of cells that respond to TGF-ß1. We were able to detect elevated furin protein levels in all tested cell lines thereby suggesting that the TGF-ß1-mediated transcriptional regulation of furin is a common feature in cell culture model systems. Importantly, SARS-CoV-2 permissive cell lines, especially Huh-7 and Calu-3, even displayed a better responsiveness to TGF-ß1 stimulation than non-permissive cell lines. This strongly indicates that blocking TGF-ß1 downstream signaling could be a suitable way to interfere with furin expression in SARS-CoV-2 infected cells. This assumption was further corroborated by another experiment showing that treatment with increasing concentrations of SB431542, the ALK5 inhibitor identified herein as antiviral compound, results in a dose-dependent decrease of furin expression. Again, the observed effects were quite different. Huh-7 and Calu-3 revealed the highest dependence on ALK5 for furin expression, while the other cells lines displayed milder reductions. One explanation might be that furin expression in Huh-7 and Calu-3 cells is exclusively regulated by Smad transcription factors that are activated by ALK5 [31,32]. In contrast, other cell lines might utilize different pathways to regulate their furin expression. Besides Smads, additional transcription factors, e.g., C/EBP-β, GATA-1, STAT-4, or HIF-1, have been shown to regulate the *furin* P1 promoter [32,75,76,77]. Cell line dependent variations in furin protein stability might be another explanation for the divergence in furin expression after ALK5 inhibition. An experiment combining SB431542 and TGF-ß1 treatment revealed that SB431542 directly acts on ALK5 to prevent TGF-ß1-mediated furin upregulation, thereby clarifying the mode of action. As elevated TGF-ß levels are a hallmark of severe COVID-19, we were able to additionally demonstrate that ALK5 inhibition also represents a therapy option under conditions with high TGF-ß1 levels [41,78,79].

By utilizing direct furin inhibitors, it has been shown that furin is essential for the efficient proteolytic processing of SARS-CoV-2 spike protein [5,6,19]. Moreover, furin activity is also related to syncytia formation and the infection of lung cells, which are both hallmarks of severe COVID-19 [5,6,13,18,19,20]. Therefore, it was an interesting finding that the delta variant, associated with severe COVID-19 and high mortality, displays two-fold better spike cleavage, and the omicron variant, associated with milder cases, two-fold poorer spike cleavage than the original Wuhan strain. In accordance, enhanced syncytia formation and pathogenicity has been recently observed for the delta variant, while the omicron variant displayed less efficient cell–cell fusion as well as attenuated replication and pathogenicity [18,64,80,81,82,83,84]. A further study suggests that the P681R mutation in the multibasic cleavage site is responsible for the success of the delta variant as it is accompanied by an enhanced fusogenicity and pathogenicity [84]. The multibasic cleavage site of omicron contains three mutations (H655Y, N679K, and P681H) that are individually predicted to favor spike S1/S2 cleavage [85]. However, we and others were able to show that the observed cleavage of spike was lower compared to the Wuhan and delta [64,80]. It is already known that spike cleavage by furin at the S1/S2 site facilitates cleavage by TMPRSS2. In accordance, the inefficient TMPRSS2-usage of omicron and decreased entry into cells with high TMPRSS2 expression (like Calu-3 and Caco-2) and lower airway organoids with high TMPRSS2 expression has been observed, while entry into cells with low TMPRSS2 expression was comparable to Wuhan and delta [64,80,81]. Thus, mutations in the multibasic cleavage site, which are associated with decreased spike cleavage, are an explanation for the milder disease caused by omicron. This led us to suggest that an efficient cleavage by furin is a prerequisite for the development of severe COVID-19, emphasizing the need for therapeutics targeting furin.

Having shown that ALK5 inhibition leads to attenuated furin levels, in the next experiment we evaluated the effects on spike cleavage. By utilizing the ALK5 inhibitors SB431542 and Vactosertib, an inhibitor currently undergoing clinical trials, we observed a decreased spike cleavage in HEK293T cells. The milder effects of the ALK5 inhibitors in comparison to the direct furin inhibitor CMK can be attributed to the fact that furin levels were only two-fold decreased after ALK5 inhibition in HEK293T cells. Residual amounts of cleaved spike in cells treated with 10 µM CMK were also observed in another study and are presumably caused by an incomplete inhibition of its enzymatic activity, as CMK at 50 µM has been demonstrated to completely block spike cleavage [6,19].

Spike-mediated cell–cell fusion, which requires the efficient processing of spike by furin, is the initial step for the formation of syncytia that facilitates viral dissemination, cytopathicity, immune evasion, and inflammatory response, thus probably contributing to pathology [86]. To assess spike-mediated cell–cell fusion under ALK5 inhibition, we adopted a protocol from the Lu group [65]. According to our spike cleavage studies, we observed significantly attenuated spike-mediated cell–cell fusion by both SB431542 and Vactosertib, indicating that both drugs may efficiently interfere with cell–cell mediated viral dissemination. Finally, our experiments with live virus demonstrated the reduced infectivity of the newly produced SARS-CoV-2 particles upon treatment with SB431542.

Nevertheless, we are aware that proteases other than furin might be involved in spike processing and subsequent cell–cell fusion, thereby exposing the possible limitations of ALK5 inhibition as a therapy option. At least PC1, a proprotein convertase also targeted by CMK, as well as trypsin, matriptase, cathepsin B, and cathepsin L have been shown to cleave synthetic substrates mimicking the multibasic cleavage site [87]. Moreover, studies using pseudoviruses and selective protease inhibitors suggest that alternative proteases can cleave at the multibasic cleavage site in cells [88]. However, the relevance of these proteases during SARS-CoV-2 infection needs to be determined.

**Figure 8:**
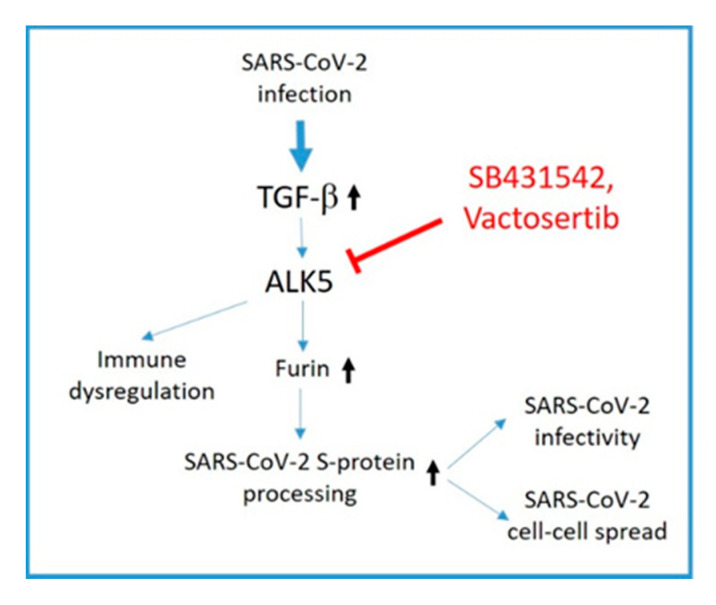
ALK5 inhibition as a potential therapy option for SARS-CoV-2 infection. SARS-CoV-2 infection leads to upregulation of TGF-β and subsequent ALK5 activation. As shown in this study, ALK5 activation induces furin expression which fosters SARS-CoV-2 S-protein processing. This increases SARS-CoV-2 infectivity and cell–cell spread. ALK5 inhibition by SB431542 or Vactosertib interferes with furin upregulation and may also block TGF-β driven immune dysregulation.

In this study, we demonstrate that ALK5 inhibition results not only in a decrease in furin expression, which was dramatic in permissive cell lines like Huh-7 and Calu-3, but also in attenuated spike cleavage and cell–cell fusion leading to a reduced production of infectious progeny SARS-CoV-2. In this context, it is noteworthy that a positive feedback loop exists between furin and TGF-ß, as furin is the proprotein convertase required to process the TGF-ß precursor into its mature form [89]. Thus, the effects of ALK5 inhibitors might accumulate over time, since furin is transcriptionally regulated by mature TGF-ß [30]. In addition, ALK5 inhibitors might be more potent under conditions with high TGF-ß levels, e.g., severe COVID-19. Moreover, by applying ALK5 inhibitors, we would expect an inhibiting dual effect on the TGF-ß-dominated cytokine storm, as both TGF-ß maturation and TGF-ß downstream signaling are affected by ALK5 inhibition (Figure 8). Apart from TGF-ß, furin processes another SARS-CoV-2 related factor, the insulin-like growth factor-1 receptor (IGF-1R) [90,91]. Interestingly, IGF-1R is considered to be related to the development of ARDS, a syndrome induced by the cytokine storm [92].

Of note, several ALK5 inhibitors are presently in preclinical or clinical studies. While cardiovascular toxicities have been observed in preclinical models, ALK5 inhibitors undergoing clinical trials neither showed such adverse effects nor any other severe side effects and have been proven safe and well-tolerated [93,94,95,96,97]. Thus, a combined use of ALK5 inhibition, interfering with cell–cell spread, and the application of neutralizing monoclonal antibodies, which targets cell-free infection, might represent a suitable therapy option in severe COVID-19.

## 5. Conclusions

In conclusion, we propose the inhibition of ALK5 as a potential therapy option for SARS-CoV-2-related disease, particularly severe COVID-19. As demonstrated in this study, the targeting of ALK5 leads to a downregulation of furin, which is most pronounced in the presence of high levels of TGF-β. This attenuates viral infectivity and interferes with syncytia formation, a hallmark of COVID-19 lung pathology. Furthermore, ALK5 inhibition may also prevent additional pathologies induced by the TGF-β dominated cytokine storm of severe COVID-19. The availability of ALK5 inhibitors undergoing phase II trials for cancer therapy should facilitate clinical studies investigating the impact of ALK5 interference on the outcome of COVID-19.

## Data Availability

Not applicable.

## Supplementary Materials

The following supporting information can be downloaded at: https://www.mdpi.com/article/10.3390/v14061308/s1, Figure S1: Screening of kinase inhibitors against SARS-CoV-2; Figure S2: SB431542 is cytotoxic at concentrations higher than 25 µM; Table S1: List of kinase inhibitors and corresponding targets used for screening.
