# Supplementary material for: Inhibitors of Activin Receptor-like Kinase 5 Interfere with SARS-CoV-2 S-Protein Processing and Spike-Mediated Cell Fusion via Attenuation of Furin Expression
Source: Viruses. 2022 Jun 15;14(6):1308. doi: 10.3390/v14061308 (PMC9228453; doi:10.3390/v14061308)
Supplement: Supplementary file 1 [file viruses-14-01308-s001.zip › viruses-1762992-supplementary.pdf]

viruses MDPI

Supplementary Materials

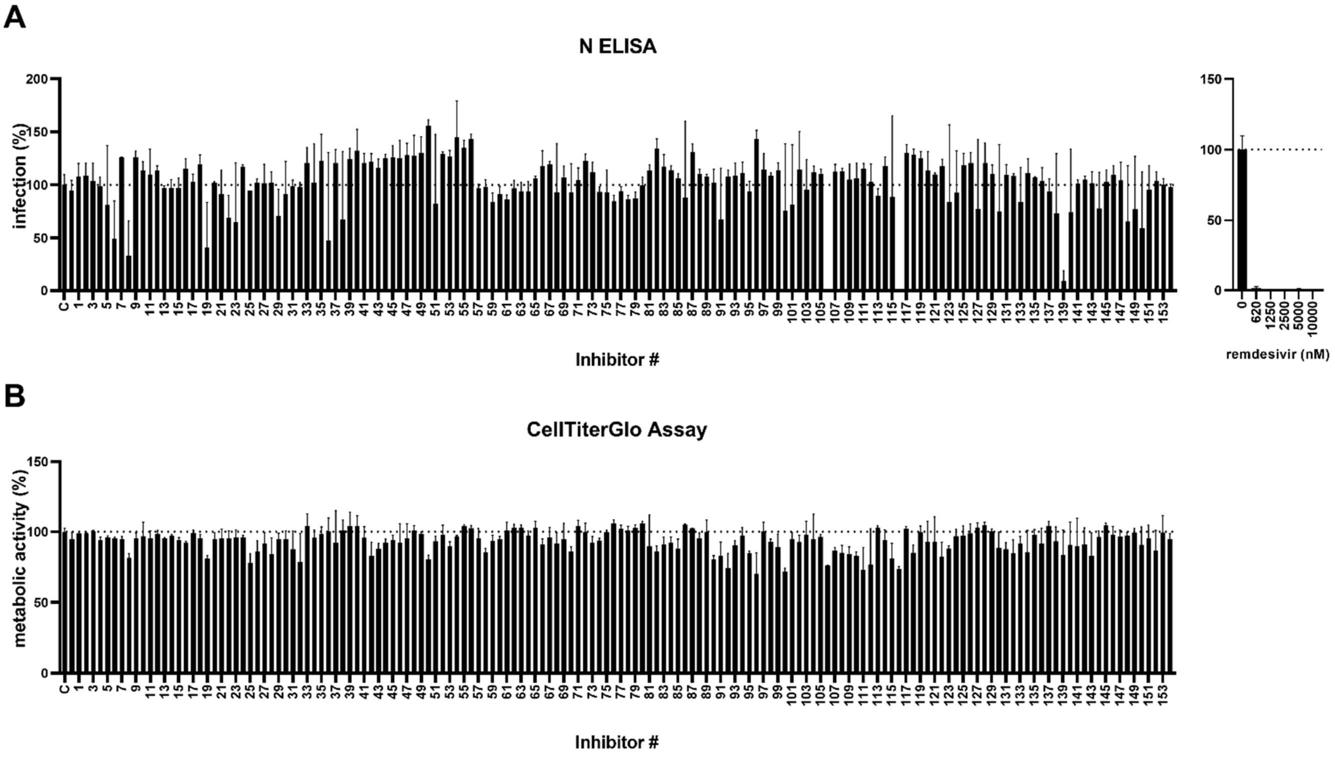

Supplementary Figure S1. Screening of kinase inhibitors against SARS-CoV-2. (**a**) Compounds were prediluted in medium and added onto Caco-2 cells (final conc. 1 µM). Additionally, a titration series of the control inhibitor remdesivir was applied. Then, cells were infected with SARS-CoV-2 Alpha at a MOI of 0.0005. Two days later, infection was quantified by an in-cell ELISA which enzymatically quantifies nucleocapsid protein. Values were corrected for the background signal derived from uninfected cells and untreated controls were set to 100% infection. (**b**) In parallel, a viability assay was performed under conditions corresponding to the respective infection assay. After two days, metabolic activity of the cells was analyzed using the CellTiter-Glo® Luminescent Cell Viability Assay. Untreated controls were set to 100% viability. Shown are means ± SD from one experiment in triplicates.

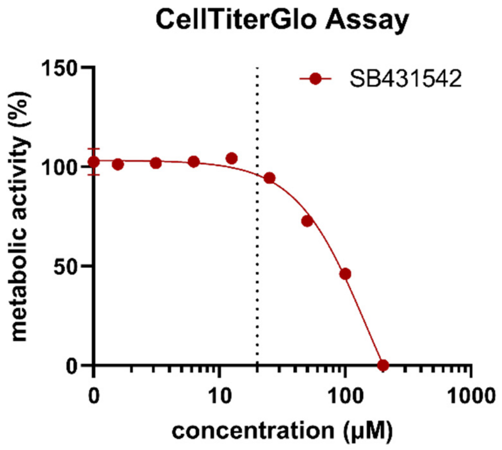

Supplementary Figure S2. SB431542 is cytotoxic at concentrations higher than 25 µM. Calu-3 cells were treated with SB431542. Metabolic activity was determined two days post infection by CellTiterGlo assay. Values were normalized to untreated controls. Data are derived from one experiment performed in triplicates and represent are mean values ± SD. Dotted line indicates highest concentration used in infection experiments (see Figure 7).

Supplementary Table S1: List of kinase inhibitors and corresponding targets used for screening

#	Inhibitor	Target
**1**	TG003	CLK1<br>CLK4
**2**	PKC 412	Multiple Kinases
**3**	Doramapimod	p38 MAPK
**4**	Erlotinib	EGFR
**5**	PLX4032	B-RAF<br>B-RAFV600E
**6**	Phthalazinone pyrazole	Aurora A
**7**	AG-879	BMX
**8**	Torin 1	mTOR
**9**	BIBF 1120	FGFR<br>PDGFR<br>VEGFR
**10**	SMI-4a	PIMs
**11**	AS-703026	MEK1<br>MEK2
**12**	Chelerythrine (chloride)	PKC
**13**	R406	Syk
**14**	AZD 7762	Chk1<br>Chk2
**15**	Dasatinib	Abl<br>LCK<br>Src
**16**	GSK1059615	PI3Kα
**17**	Ruxolitinib	JAK1<br>JAK2
**18**	Necrostatin-1	RIPK1
**19**	INK128	mTOR
**20**	Bosutinib	Abl<br>EPHB2<br>Src
**21**	Canertinib (hydrochloride)	EGFR
**22**	(R)-Crizotinib	ALK<br>c-MET
**23**	SB-431542 (hydrate)	ALK5
**24**	PD 173074	FGFR1
**25**	PD 0325901	MEK
**26**	SB 203580	p38 MAPK
**27**	VX-702	p38 MAPK
**28**	Emodin	CK2

**29**	CHIR99021	GSK3α<br>GSK3β
**30**	BIO	GSK3α<br>GSK3β
**31**	Imatinib (mesylate)	Multiple Kinases
**32**	Sunitinib (malate)	FLK1<br>FLT3<br>PDGFRβ
**33**	PP2	Fyn<br>HCK<br>LYN
**34**	3-Methyladenine	PI3K
**35**	Bisindolylmaleimide I	PKC
**36**	Bisindolylmaleimide V	S6K
**37**	D 4476	CK1
**38**	Gö 6983	PKC
**39**	H-9 (hydrochloride)	PKG
**40**	Indirubin-3'-monoxime	GSK3β
**41**	NU 6102	Cdk1<br>Cdk2
**42**	KN-62	CAMKII
**43**	KN-93	CAMKII
**44**	CGP 57380	MNK1
**45**	(S)-Glycyl-H-1152 (hydrochloride)	ROCK-II
**46**	Bisindolylmaleimide IX (mesylate)	GSK3<br>PKC
**47**	SU6656	Yes
**48**	LY364947	ALK5
**49**	CAY10621	SPHK1
**50**	YM-201636	PIKfyve
**51**	ZM 447439	Aurora B
**52**	AS-041164	PI3Kγ
**53**	NVP-AEW541 (hydrochloride)	IGF-1R
**54**	PP242	mTOR
**55**	ABT-869	PDGFR family<br>VEGFR family
**56**	CAY10622	ROCK-I<br>ROCK-II
**57**	17β-hydroxy Wortmannin	PI3K
**58**	CAY10626	mTOR<br>PI3Kα

**59**	SU 6668	Aurora B
**60**	PHA-665752	c-Met
**61**	BX-912	PDK1
**62**	Vatalanib (hydrochloride)	VEGFR family
**63**	KRN 633	VEGFR family
**64**	LCK Inhibitor	LCK
**65**	BI-D1870	RSKs
**66**	CRT0066101 (hydrochloride)	PKD
**67**	GNE-7915	LRRK2
**68**	GNF-5	Bcr-Abl
**69**	BMS 345541 (trifluoroacetate salt)	IKKα<br>IKKβ
**70**	NVP-TAE226	FAK<br>PYK2β
**71**	AZ191	DYRK1B
**72**	Tie2 Kinase Inhibitor	Tie2
**73**	Afuresertib (hydrochloride)	PKB/Akt
**74**	GSK2334470	PDK1
**75**	JNK Inhibitor XVI	JNK1<br>JNK2<br>JNK3<br>JNKs
**76**	BI-6727	Plks
**77**	Syk Inhibitor II	Syk
**78**	PF-06463922	ALK
**79**	PF-562271 (besylate)	FAK
**80**	BMS-777607	Axl family<br>Met family
**81**	URMC-099	LRRK2<br>MLKs
**82**	BGJ398	FGFR family
**83**	Mps1-IN-1	Mps1
**84**	WZ4003	NUAK1<br>NUAK2
**85**	MLCK Inhibitor Peptide 18	MLCK
**86**	AP26113	ALK
**87**	AZ 3146	Mps1
**88**	AZD 1208	PIMs
**89**	RPI-1	RET
**90**	LDN-211904	EphB3

**91**	BMS-5	LIMK1<br>LIMK2
**92**	TAS 120	FGFR family
**93**	Apatinib	VEGFR2
**94**	ARQ-092	PKB/Akt
**95**	LY2606368	Chk1
**96**	FRAX597	PAK1<br>PAK2<br>PAK3
**97**	KW 2449	Abl<br>FLT3
**98**	UNC569	Axl family
**99**	XMD16-5	TNK2
**100**	VE-822	ATR
**101**	Tilfrinib	BRK/PTK6
**102**	AZD 0156	ATM
**103**	LOXO-101	Trk family
**104**	LOXO-195	Trk family
**105**	U-0126	MEK
**106**	Staurosporine	PKC
**107**	Y-27632 (hydrochloride)	ROCKs
**108**	Leelamine	PDK
**109**	PD 169316	p38 MAPK
**110**	TGX-221	PI3K p110β
**111**	(S)-H-1152 (hydrochloride)	ROCKs
**112**	AS-605240	PI3Kα,β,δ,γ<br>PI3Kγ
**113**	JNJ-10198409	PDGFR
**114**	CAY10505	CK2
**115**	PI-103	DNA-PK<br>mTOR<br>PI3K
**116**	PIK-75 (hydrochloride)	PI3K p110α
**117**	Sphingosine Kinase Inhibitor 2	SPHK1
**118**	SC-1	Stem Cell Renewal
**119**	(R)-Roscovitine	CDKs
**120**	Sorafenib	Multiple Kinases
**121**	CAY10561	ERK2
**122**	PI3-Kinase α Inhibitor 2	PI3Kα
**123**	ML-9	Multiple Kinases
**124**	Erbstatin analog	EGFR

**125**	Kenpaullone	CDKs<br>GSK3β
**126**	Olomoucine	CDKs
**127**	AG-494	EGFR
**128**	AG-825	ErbB2
**129**	AG-1478	EGFR
**130**	SB-216763	GSK3
**131**	H-8 (hydrochloride)	PKA<br>PKG
**132**	LFM-A13	BTK
**133**	SC-514	IKKβ
**134**	RG-13022	EGFR
**135**	AG-490	JAK2
**136**	AG-183	EGFR
**137**	Lavendustin C	EGFR
**138**	GW 5074	Raf-1
**139**	5-Iodotubercidin	CK1<br>ERK2<br>PKC
**140**	SB 202190	p38 MAPK
**141**	CAY10571	p38α MAPK
**142**	Nilotinib	Bcr-Abl
**143**	SP 600125	JNKs
**144**	H-89 (hydrochloride)	Multiple Kinases
**145**	HA-1077 (hydrochloride)	ROCK-II
**146**	AG-370	PDGFR
**147**	Wortmannin	PI3K<br>Plk1<br>Plk3
**148**	AG-1296	PDGFR
**149**	WHI-P131	JAK3
**150**	CAY10574	Cdk9
**151**	CAY10576	IKKε
**152**	TWS119	GSK3β
**153**	CAY10578	CK2
**154**	PD 184161	MEK1<br>MEK2
